# Increased Protein Stability and Interleukin-2 Production of a LAT^G131D^ Variant With Possible Implications for T Cell Anergy

**DOI:** 10.3389/fcell.2020.561503

**Published:** 2020-09-11

**Authors:** Mikel M. Arbulo-Echevarria, Inmaculada Vico-Barranco, Isaac Narbona-Sánchez, Francisco García-Cózar, Arkadiusz Miazek, Enrique Aguado

**Affiliations:** ^1^Institute of Biomedical Research Cadiz (INIBICA), Cádiz, Spain; ^2^Department of Biomedicine, Biotechnology and Public Health (Immunology), University of Cádiz and Puerto Real University Hospital Research Unit, Cádiz, Spain; ^3^Department of Biochemistry and Molecular Biology, Wrocław University of Environmental and Life Sciences, Wrocław, Poland

**Keywords:** LAT, TCR, phosphorylation, anergy, IL-2 (interleukin-2)

## Abstract

The adaptor LAT plays a crucial role in the transduction of signals coming from the TCR/CD3 complex. Phosphorylation of some of its tyrosines generates recruitment sites for other cytosolic signaling molecules. Tyrosine 132 in human LAT is essential for PLC-γ activation and calcium influx generation. It has been recently reported that a conserved glycine residue preceding tyrosine 132 decreases its phosphorylation kinetics, which constitutes a mechanism for ligand discrimination. Here we confirm that a LAT mutant in which glycine 131 has been substituted by an aspartate (LAT^G131D^) increases phosphorylation of Tyr132, PLC-γ activation and calcium influx generation. Interestingly, the LAT^G131D^ mutant has a slower protein turnover while being equally sensitive to Fas-mediated protein cleavage by caspases. Moreover, J.CaM2 cells expressing LAT^G131D^ secrete greater amounts of interleukin-2 (IL-2) in response to CD3/CD28 engagement. However, despite this increased IL-2 secretion, J.CaM2 cells expressing the LAT^G131D^ mutant are more sensitive to inhibition of IL-2 production by pre-treatment with anti-CD3, which points to a possible role of this residue in the generation of anergy. Our results suggest that the increased kinetics of LAT Tyr132 phosphorylation could contribute to the establishment of T cell anergy, and thus constitutes an earliest known intracellular event responsible for the induction of peripheral tolerance.

## Introduction

After the specific recognition of a peptide antigen bound to an MHC molecule on the surface of an Antigen Presenting Cell, a cascade of intracellular signaling events are triggered in T lymphocytes (Malissen and Bongrand, 2015; Alcover et al., 2018; Courtney et al., 2018). The transmembrane adaptor LAT (Linker for the Activation of T cells) constitutes a major ZAP-70 substrate, and initiates most of the intracellular events that characterize the T cell receptor (TCR) signaling pathway (Weber et al., 1998; Zhang et al., 1998). Early on after initial LAT cloning and characterization, experiments performed with the J.CaM2 LAT-deficient T cells analyzed the contribution of some of the nine conserved LAT tyrosines to the TCR/CD3 signaling cascade (Finco et al., 1998; Zhang et al., 2000; Lin and Weiss, 2001; Paz et al., 2001). Those early works showed that the three most distal tyrosines (171, 191 and 226 in the human form of LAT) are necessary for Grb2-SOS and Gads-SLP76 binding and Erk activation and that the sixth tyrosine residue (132 in human LAT) is essential for PLC-γ1 binding and activation and calcium influx generation. Moreover, the phenotype of LAT-knockout (KO) mice revealed the essential role of this molecule for the transduction of intracellular signals emanating from the pre-TCR, since thymic development was completely blocked at the CD4-CD8- Double Negative (DN) stage (Zhang et al., 1999).

Therefore, TCR engagement triggers the assembly of a LAT signalosome linking the TCR to activatory signaling pathways that govern T-cell development and activation (Malissen et al., 2005). However, two LAT-knockin (KI) strains of mice harboring point mutations in the four most distal tyrosines developed lymphoproliferative disorders involving helper *T* (*T*_H_) cells (Aguado et al., 2002; Sommers et al., 2002; Nunez-Cruz et al., 2003). The analysis of those mice strains revealed for the first time that the LAT adaptor acts not only as a transducer of activation signals, but also constitutes a negative regulator of TCR signaling and T-cell homeostasis. One of these strains of mice had a Tyr to Phe mutation in tyrosine 136 of LAT (mouse ortholog of human tyrosine 132, LAT^Y136F^ KI), and presented a paradoxical phenotype with a lymphoproliferative disorder of polyclonal CD4 T cells along with high Th2 cytokine production, despite a reduction in thymic development (Aguado et al., 2002; Sommers et al., 2002). The phenotype of these mice constituted the first evidence of a special inhibitory function for LAT, mainly played by the sixth tyrosine residue.

The order of signaling events taking place after TCR engagement is critical for productive immune responses. Lck initiates intracellular signaling in T cells by phosphorylating CD3 and ζ-chain ITAMs, leading to ZAP-70 recruitment and activation (Chan et al., 1991; Wange et al., 1993; Iwashima et al., 1994). Once activated ZAP-70, and not Lck, phosphorylates LAT and SLP-76 tyrosines by utilizing an electrostatic mechanism that favors phosphorylation of tyrosines surrounded by negatively charged residues, and excludes phosphorylation of tyrosines close to positively charged amino acids (Shah et al., 2016, 2018). Tyrosine residues 127, 171, 191, and 226 of human LAT are all preceded by aspartic or glutamic acid residues, and, as shown by means of an approach using bacterial surface-display, cell sorting, and deep sequencing, these tyrosines are efficiently phosphorylated by ZAP-70. However, tyrosine 132 of LAT is preceded by a well-conserved glycine residue, which should preclude its efficient phosphorylation, and so PLC-γ activation, calcium influx generation, and, ultimately, the activation of T lymphocytes. Indeed, in agreement with such a hindrance, in both Jurkat cells and primary human T cells, the kinetics of tyrosine 132 phosphorylation of LAT is much slower than the one of tyrosine 191 (Houtman et al., 2005). This is bewildering, given the essential role of tyrosine 132 phosphorylation for complete T cells activation (Zhang et al., 2000; Lin and Weiss, 2001; Paz et al., 2001; Aguado et al., 2002; Sommers et al., 2002). Recent work has shown that glycine 131 mutation by an aspartate residue in LAT increases LAT-Y132 phosphorylation, but not the one of tyrosines 171, 191 or 226 (Lo et al., 2019). Moreover, more distal signaling events are also increased in cells expressing the LAT^G131D^ mutant, for example, PLC-γ phosphorylation, Ca^2+^ influx, Erk phosphorylation or CD69 expression. Interestingly, Lo et al. demonstrate in this work that cells expressing the LAT^G131D^ mutant responded with greater intensity to lower anti-CD3 concentrations than did wild-type cells, and the same behavior was observed for low-affinity ligands in Jurkat cells expressing the OTI TCR, or primary T cells from a mouse strain expressing a floxed *Lat* allele, which allowed authors to delete endogenous LAT expression and express wild-type LAT or a LAT^G131D^ mutant. Lentiviral expression in mouse primary cells of a LAT^G131D^ mutant also increased the production of IFN-γ, which constitutes a piece of evidence that the brake imposed by Gly 131 has effects in the final activation of T lymphocytes. However, Weiss and collaborators did not analyze the production of IL-2 in either Jurkat cells or primary cells. This is of relevance since the increase in calcium responses shown by cells expressing LAT^G131D^ may induce a greater production of this cytokine.

In the present report, we analyze the effects of expressing a LAT^G131D^ mutant in the J.CaM2 LAT deficient cell line. We verify the findings of Lo et al., showing that this LAT mutant induces increased tyrosine phosphorylation of LAT specifically at residue 132, increased phosphorylation of PLC-γ and Ca^2+^ responses after CD3 stimulation. Moreover, we observe an increase in LAT protein stability, despite normal Fas-mediated cleavage, and augmentation of IL-2 production after CD3/CD28 cross-linking. Interestingly, J.CaM2 cells expressing the LAT^G131D^ mutant are more sensitive to inhibition of IL-2 production by pre-treatment with anti-CD3, which points to a possible role of this residue in the generation of anergy.

## Method

### Antibodies and Reagents

The anti-Fas (IgM) antibody was from Merck-Millipore; anti-LAT LAT-01 mAb was from EXBIO (Praha, Czech Republic); anti-LAT 11B.12, anti-PLC- γ, anti-PTP1B, and anti-caspase-3 monoclonal antibodies were from Santa Cruz Biotechnology (Heidelberg, Germany); antibodies binding phospho-Erk, β-actin, phospho-PLC-γ1-Tyr783 and phospho-LAT-Tyr171 were from Cell Signaling Technology; anti-6His-HRP antibody and anti-phospho-LAT-Tyr132 were from Abcam (Cambridge, United Kingdom). The protein synthesis inhibitor cycloheximide was purchased from Merck-Millipore. Stimulations were performed with the anti-human CD3 OKT3 monoclonal antibody (eBioscience).

### Enzyme−Linked Immunosorbent Assay (ELISA) and Anergy Induction

IL-2 release from lentivirally transduced J.CaM2 cells was measured by human IL−2 ELISA set (MAX Standard, Biolegend, Fell, Germany), using 96−well Nunc MaxiSorp microtiter plates. Supernatants of resting or activated cells were analyzed in comparison to a standard curve of IL-2. Absorbance was determined using a Synergy MX Multi-Mode Reader (Biotek, Bad Friedrichshall, Germany) set to 405 and 450 nm. For anergy induction, 24 well plates were coated overnight at 4°C with 10, 2, 0.5, or 0.2 μg/ml OKT3 mAb in Tris 0.1 M buffer (pH 8.2; 200 μl/well). After washing three times with phosphate−buffered saline (PBS), cells expressing wild-type or the mutant form of LAT were incubated overnight and then stimulated in 96 well plates with anti-CD3/CD28 microbeads (at a bead-to-cell ratio of 3:1) for 48 h. Supernatants were analyzed by the human IL−2 ELISA set.

### Cell Culture

The LAT-deficient J.CaM2 cell line was generously provided by Dr. Arthur Weiss, University of California, San Francisco (CA, United States). Cells were grown in complete RPMI 1640 medium (Lonza) supplemented with 10% FCS (Lonza) and 2 mM L-glutamine at 37°C in a humidified atmosphere containing 10% CO^2^.

### Mutagenesis and Lentiviral Transduction

LAT cDNA cloning was performed as previously described (Garcia-Blesa et al., 2013). Site-directed mutagenesis was performed to change the sequence coding for glycine 131. Coding sequences in the plasmids were verified by sequencing and then subcloned in frame with GFP in the SIN lentiviral transfer plasmid pHR’SINcPPT-Blast through site-specific recombination (Gateway LR Clonase, Invitrogen). Lentiviral supernatants were generated as previously described (Garcia-Blesa et al., 2013) and used to induce expression of WT-LAT or the LAT-NIL mutant in J.CaM2 cells. Blasticidin selection (20 μg/ml) was applied to transduced cells after 72 h of culture, and the expression of GFP was analyzed using FACS analysis (CytoFLEX, Beckman Coulter).

### Preparation of Cell Lysates and Western Blotting

Lentivirally transduced J.CaM2 cells were starved in RPMI 1640 without FCS for 18 h before being stimulated with anti-CD3 mAb at 37°C. Cells were then lysed at 2.0 × 10^7^ cells/ml in 2X Laemmli buffer, followed by incubation at 99°C for 5 min and sonication. For anti-Fas stimulation, cells were incubated with 100 ng/ml of anti-Fas mAb at 1 × 10^6^ cells/ml in RPMI 1640, supplemented with 10% FCS, and then pelleted and lysed as described above. For Western blotting, whole-cell lysates were separated by SDS-PAGE and transferred to PVDF membranes, which were incubated with the indicated primary antibodies, followed by the appropriate secondary antibody conjugated to IRDye 800CW (Li-Cor, Lincoln, NE, United States) or horseradish peroxidase (HRP). Reactive proteins were visualized using the Odyssey CLx Infrared Imaging System (Li-Cor) or by enhanced chemiluminescence (ECL) acquired in a ChemiDoc Touch Imaging System (Bio-Rad Laboratories). For reprobing, PVDF membranes were incubated for 10 min at room temperature with WB Stripping Solution (Nacalai Tesque, Kyoto, Japan), followed by a TTBS wash. For the cycloheximide chase assay, cells were treated with 0.1 mM cycloheximide for up to 10 h. At the indicated time points, cell samples were obtained and lysed in 2X Laemmli buffer, and LAT protein levels were determined by immunoblotting and quantified by densitometry.

### Ca^2+^ Mobilization

Measurement of intracellular free Ca^2+^ was carried out using Indo-1 AM (acetoxymethyl) (2 μM; Molecular Probes, Invitrogen) as previously described (Garcia-Blesa et al., 2013). Calcium measurements were performed using a Synergy MX Multi-Mode Reader (Biotek) at 37°C. Cells were excited by light at a wavelength of 340 nm, and the fluorescence emitted at 405 and 485 nm was collected alternately per second. Calcium mobilization was evaluated by the ratio of 405/485 nm fluorescence signal.

### Statistical Analysis

Western blots were densitometrically quantified, and statistics were performed with Microsoft Excel using a two-tailed *t*-test. Levels of significance *p* < 0.05 are presented as ^∗^.

## Results and Discussion

### Generation of Lentiviral Transfectants of J. CaM2 Cells Expressing Wild-Type LAT and the LAT^G131D^ Mutant

To study the role of the conserved glycine residue preceding tyrosine 132 in LAT we generated lentiviral plasmids to express wild-type LAT or a LAT^G131D^ mutant in J.CaM2 cells, as previously described (Arbulo-Echevarria et al., 2016, 2018). This would allow us to verify the effects observed by Lo et al. (2019). in Jurkat cells in which CRISPR was performed to eliminate the *Lat* gene in both chromosomes, and also address other questions of interest. We designed lentiviral vectors containing the coding region of wild-type or the mutant LAT fused to a 6-His tag, followed by an IRES sequence and a GFP reporter (Supplementary Figure S1A). Lentiviral supernatants were generated and used for infection of J.CaM2 cells obtaining transfection levels always greater of 75% of cells, as measured by GFP expression (Supplementary Figure S1B). GFP levels were always similar in cells expressing wild-type LAT and the LAT^G131D^ mutant, although sometimes levels of GFP were slightly higher in J.CaM2 cells expressing wild-type LAT. To assure that any possible difference in the responses observed in cells expressing WT-LAT and LAT^G131D^ mutant was not due to differential expression of the TCR/CD3 complex, we analyzed by flow cytometry CD3 expression. As shown in Supplementary Figure S1B, CD3 expression was indistinguishable in J.CaM2 cells expressing WT-LAT and the LAT^G131D^ mutant. Next, to determine LAT levels in lentivirally transduced cells, Western blot analysis was performed with non-transduced JCaM2 cells or expressing WT-LAT or LAT^G131D^ mutant. As it can be seen in Supplementary Figure S1C, Western blot performed with the mAb LAT01 did not detect LAT expression in J.CaM2 cells transduced with the LAT^G131D^ mutant (Supplementary Figure S1C, left panel). This result was unexpected since these cells had similar GFP expression levels than WT-LAT expressing cells. Western blots performed with an anti-6His mAb showed bands of similar intensity in both WT-LAT and LAT^G131D^ transduced cells (Supplementary Figure S1C, middle panel), supporting that the LAT^G131D^ mutant was expressed at similar levels than WT-LAT but was not recognized by the LAT01 mAb. Indeed, a different mAb (11B.12, Supplementary Figure S1C, right panel) showed similar reactivity in both types of cells. Therefore, it seems that the Gly to Asp amino acid substitution performed in the LAT^G131D^ mutant affects the epitope recognized by LAT01 mAb, and may constitute a way to differentiate wild-type LAT and the LAT^G131D^ mutant.

### Mutation of Gly 131 to Asp of LAT Increases Intracellular Signaling

**FIGURE 1:**
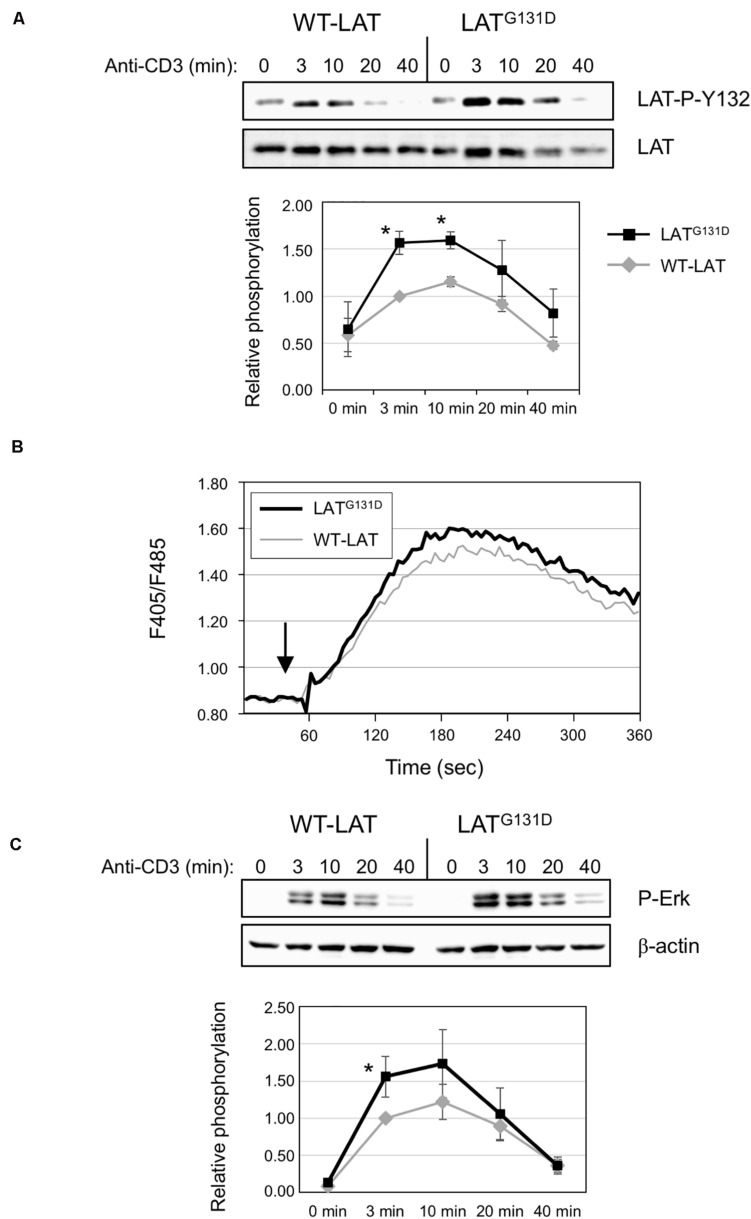
Mutation of Gly 131 to Asp increases TCR-dependent intracellular signaling. **(A)** Immunoblots analyzing phosphorylation of LAT at tyrosine residue 132 in cells stimulated with soluble anti-CD3 were done with the phospho-specific antibody. Membranes were stripped and blotted with anti-LAT antibody (middle panel). The lower diagram represents the mean fold increase in phosphorylation in four independent experiments using J.CaM2 cells expressing WT-LAT (gray line) or the LAT^G131D^ mutant (black line). Phosphorylation levels were normalized to total LAT expression. Bars represent the standard error. The asterisk represents statistical significance. **(B)** J.CaM2 cells expressing WT-LAT or the LAT^G131D^ mutant were loaded with Indo-1AM and stimulated with OKT3 mAb (1 μg/ml) at the indicated time (black arrow). The intracellular Ca^2+^ concentration was determined at 37°C through the change in Indo-1AM fluorescence. The graphic represents the average of five experiments. **(C)** Whole-cell lysates were probed by Western blotting for the activation of Erk by using a mAb recognizing doubly phosphorylated on specific threonine and tyrosine residues on Erk (upper panel). Stripped membranes were blotted with anti-β-actin mAb to show equal protein expression (middle panel). Lower diagram represents the mean fold increase in Erk phosphorylation in four independent experiments using J.CaM2 cells expressing WT-LAT (gray line) or the LAT^G131D^ mutant (black line). Phosphorylation levels were normalized to β-actin expression. Bars represent the standard error. Asterisk represents statistical significance.

To verify whether the mutation of glycine 131 to aspartate reproduced the effects observed by Lo et al. (2019), we performed anti-CD3 stimulations in WT-LAT and LAT^G131D^ expressing J.CaM2 cells. As previously reported, the substitution of glycine at position 131 with an aspartate residue increased the kinetics and intensity of phosphorylation of LAT tyrosine 132, since phosphorylation 3 and 10 min after CD3 treatment induced an statistically significant increase of phosphorylation (Figure 1A). However, phosphorylation of tyrosine 171 was similar in both WT-LAT and LAT^G131D^ expressing J.CaM2 cells, confirming the specificity of the negative effect of the glycine residue in the phosphorylation of LAT Y132 (Supplementary Figure S2A).

Next, given the specific increase in phosphorylation of LAT Y132 after CD3 stimulation, we wondered if this would transduce enhanced downstream signals. Therefore, we analyzed the effect of replacing G131 by an aspartate residue on PLC-γ1 activation, which can be monitored by the phosphorylation of its tyrosine residue 783 (Wang et al., 1998). As can be seen in Supplementary Figure S2B, anti-CD3 treatment induced increased phosphorylation of PLC-γ1 in LAT^G131D^ expressing J.CaM2 cells at all the time points analyzed, supporting the view that the increased kinetics of LAT Y132 phosphorylation is enough to induce augmented downstream signals. Next we analyzed whether the mutation of G131 of LAT affected calcium influx generation. Indo-1AM labeled cells were stimulated with 1 μg/ml OKT3 mAb, and Ca^2+^ influx was analyzed. Interestingly, even at high doses of anti-CD3 (1 μg/ml) the calcium response in J.CaM2 cells expressing the LAT^G131D^ mutant was slightly higher than the one observed in WT-LAT expressing cells (Figure 1B). We also performed experiments with lower doses of OKT3 mAb, to corroborate previous results showing the difference in Ca^2+^ responses between T cells expressing LAT^G131D^ and WT-LAT were greater at low doses of the antibody. As it can be seen in Supplementary Figure S2C, stimulation with 0.5 μg/ml and 0.12 μg/ml of OKT3 showed greater differences between WT-LAT and LAT^G131D^ expressing cells. Therefore, our results confirm that glycine 131 acts as a negative regulator of LAT Y132 phosphorylation, and thus of TCR signaling.

To validate the negative role of G131 residue of LAT, we also analyzed Erk phosphorylation in WT-LAT and LAT^G131D^ expressing cells (Figure 1C). Again, mutation of glycine 131 to aspartate induced increased kinetics and intensity of Erk phosphorylation, with statistical significance at the 3 min time point (Figure 1C, lower diagram). Altogether, these data confirm that the LAT^G131D^ mutation releases the brake imposed on the TCR/CD3 signaling cassette, as previously observed by Lo et al. (2019).

### Impact of Glycine 131 Substitution on LAT Cleavage and Protein Stability

**FIGURE 2:**
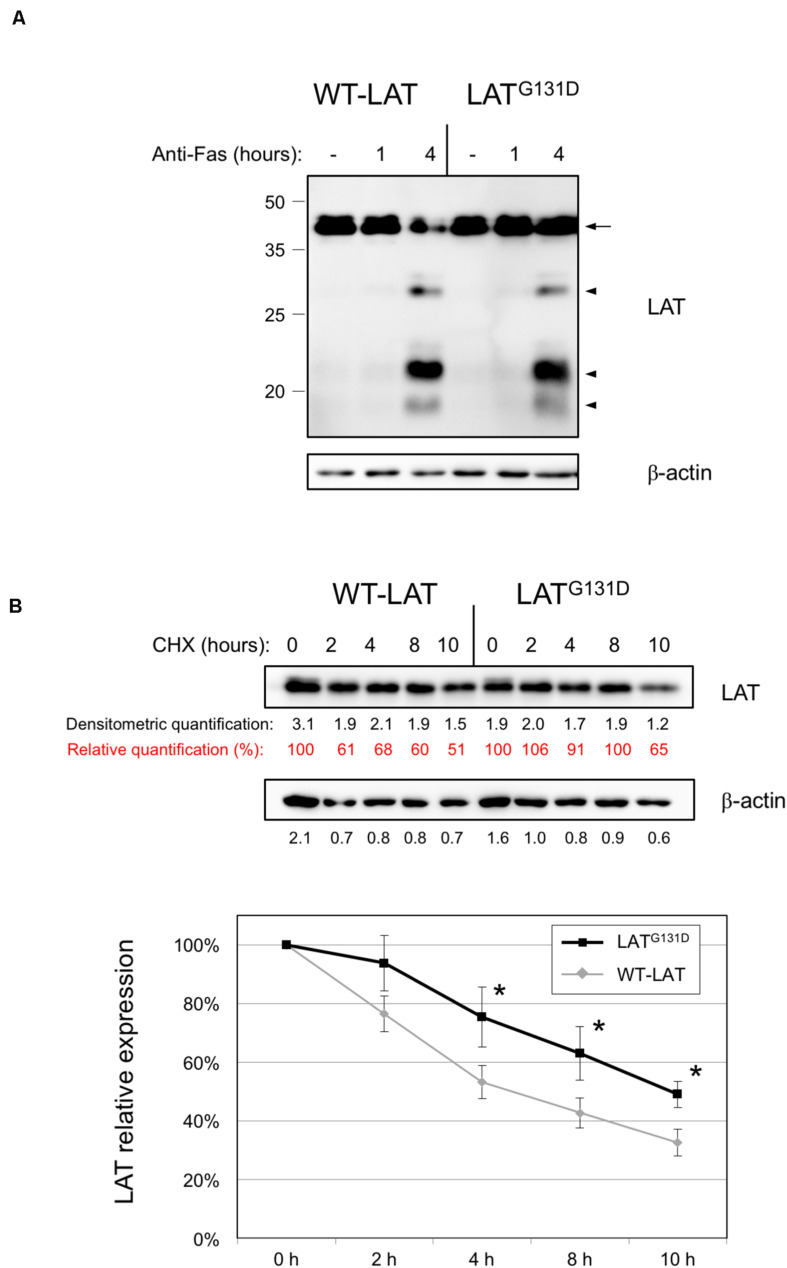
Degradation of WT-LAT and LAT^G131D^ mutant proteins. **(A)** J.CaM2 cells expressing WT-LAT or the LAT^G131D^ mutant were treated for the indicated time in hours at 37°C with 100 ng/ml of anti-Fas antibody, and LAT cleavage was assessed in total cell lysates by Western blot with an anti-LAT antibody. Molecular masses in kDa are indicated adjacent to the Western blot. Arrow indicates the band corresponding to the whole LAT molecule, and arrowheads are indicative of the proteolytic fragments. **(B)** Cell lysates obtained from J.CaM2 cells expressing WT-LAT or the LAT^G131D^ mutant, previously treated with cycloheximide (CHX) for the indicated time in hours, and LAT (upper panel) or β-actin (middle panel) protein levels were analyzed by Western blot. Black numbers below each panel represent the quantification of corresponding bands. Red numbers below LAT panel represent the percentage of LAT expression relative to the one at 0 h of cycloheximide treatment. Densitometric analysis of six experiments was performed, and the relative expression of LAT was represented (lower panel). Bars represent the standard error. Asterisks represent statistical significance.

Our group has previously demonstrated that LAT undergoes a proteolytic cleavage in T cells receiving proapoptotic stimuli (Garcia-Blesa et al., 2013; Klossowicz et al., 2013). Given that glycine in position 131 is close to one of the described cleavage points in LAT (aspartate 126), we decided to verify if the substitution of G131 by an aspartic acid residue modifies Fas-dependent LAT cleavage. Therefore, we treated lentivirally transduced J.CaM2 cells with an anti-Fas antibody for 4 h at 37°C. As it can be seen in Figure 2A, cleavage of both WT-LAT and LAT^G131D^ mutant generated two proteolytic fragments of the same electrophoretic mobility and similar intensity. Therefore, these results show that the LAT^G131D^ mutant is equally sensitive to Fas-mediated proteolytic cleavage as WT-LAT.

We have previously shown that a functional isoform of LAT originated from an intron six retention event, and that can be detected in human and other mammalian species at the RNA level, shows a shorter half-life than the canonical LAT isoform (Klossowicz et al., 2014). More recently we have described that mutation of a stretch of negatively charged residues, encoded by exon seven of human LAT, also affects LAT stability, since the substitution of this fragment with a stretch of non-charged amino acids significantly decreases LAT stability (Arbulo-Echevarria et al., 2018). Given that those LAT sequence modifications affected residues from position 113 to 126, we wondered if the substitution of glycine in position 131 of LAT would modify LAT stability. Consequently, we cultured J.CaM2 cells expressing WT-LAT or LAT^G131D^ in the presence of the translational inhibitor cycloheximide, and then cells were collected at specific time points and lysed. Surprisingly, Western blot analysis showed that the LAT^G131D^ mutant is degraded with slower kinetics than WT-LAT (Figure 2B, upper panel). Densitometric analysis of five independent experiments showed that LAT^G131D^ has increased stability in comparison with WT-LAT, with statistical significance after 4 h of cycloheximide treatment (Figure 2B, lower panel). This observation is in agreement with our previous reports about the role of this region on LAT turnover and could be, at least in part, responsible for the increase in downstream TCR-dependent signals. Overall, these results show that glycine 131 negatively influences the stability of LAT but it has no bearing on its sensitivity to Fas-mediated proteolytic cleavage.

### Cells Expressing the LAT^G131D^ Mutant Produce Greater Amounts of IL-2 After CD3/CD28 Stimulation

**FIGURE 3:**
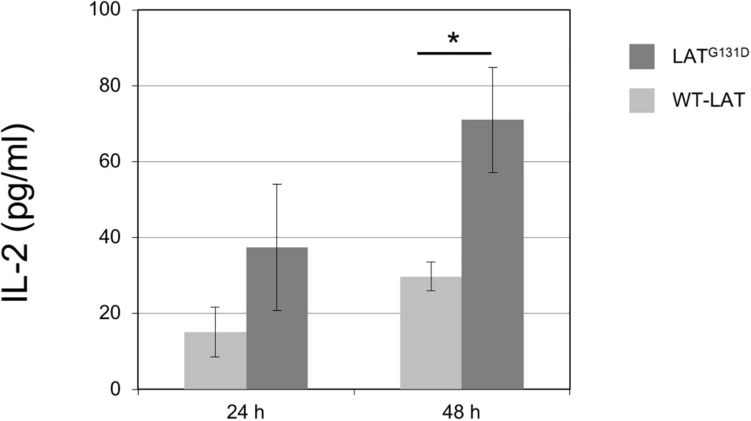
Increased capacity of IL-2 production by J.CaM2 cells expressing the LAT^G131D^ mutant. J.CaM2 cells expressing human WT-LAT or the LAT^G131D^ mutant were stimulated with anti-CD3/CD28 microbeads for the indicated time in hours, and the amount of IL-2 in supernatants was measured by ELISA. The values presented are mean values of four (for 24 h incubation) or eleven (for 48 h) separate experiments. Bars represent the standard error. The asterisk represents statistical significance.

Lo et al. (2019) have shown that mouse T cells lentivirally expressing a LAT mutant in which glycine 135 (mouse ortholog of human G131) had been substituted by an aspartate residue (LAT^G135D^ mutant) have a lower reactivity threshold to allow for IFN-γ production, and show augmented percentages of IFN-γ secreting cells. However, it remains to be determined the effect of this mutation on interleukin-2 (IL-2) secretion. IL-2 is an essential cytokine that allows controlling the differentiation and homeostasis of both pro- and anti-inflammatory T cells (Ross and Cantrell, 2018). Therefore, we cultured J.CaM2 cells expressing wild-type LAT or the LAT^G131D^ mutant for 24 and 48 h with anti-CD3/anti-CD28 beads. Supernatants were analyzed by ELISA, and the amount of IL-2 in supernatants from WT-LAT expressing cells after 24 h of CD3/CD28 stimulation was 15 ± 7 pg/ml (Figure 3), which constitutes a similar level of IL-2 production in stimulated J.CaM2 cells expressing LAT (Klossowicz et al., 2014). Interestingly, supernatants from J.CaM2 cells expressing the LAT^G131D^ mutant stimulated for 24 h contained increased levels of IL-2 levels with regard to WT-LAT expressing cells (37 ± 17 pg/ml, Figure 3), although this difference did not reach statistical significance, probably as the result of the low number of performed experiments (*n* = 4). Interestingly, analysis of supernatants from cells stimulated for 48 h with anti-CD3/anti-CD28 microbeads showed greater amounts of secreted IL-2 in both types of cells, with a statistically significant difference between WT-LAT and LAT^G131D^ expressing cells (30 ± 4 vs 71 ± 14 pg/ml, respectively; Figure 3).

However, although it has been previously shown that there is no basal expression of LAT in J.CaM2 cells, we previously reported that activation of protein kinase C (PKC) with PMA induces LAT re-expression at both mRNA and protein levels (Marek-Bukowiec et al., 2016). To rule out that endogenous LAT was not expressed, which may confound the observed results, we decided to stimulate the cells during one night with two doses of anti-CD3 immobilized to plastic, and verify by Western blot LAT expression. The endogenous form is smaller than the transfected LAT forms. As shown in Supplementary Figure S3, treatment with 2 or 10 μg/ml of OKT3 did not induce expression of the endogenous form of LAT, discarding any effect of endogenous LAT expression. Therefore, these results support a role of glycine 131 of LAT as a “brake” controlling not only early intracellular signals coming from the TCR but also late activation events that take place in fully activated T cells.

### Effect of the Gly 131 to Asp Mutation in LAT Adaptor on Anergy Induction

**FIGURE 4:**
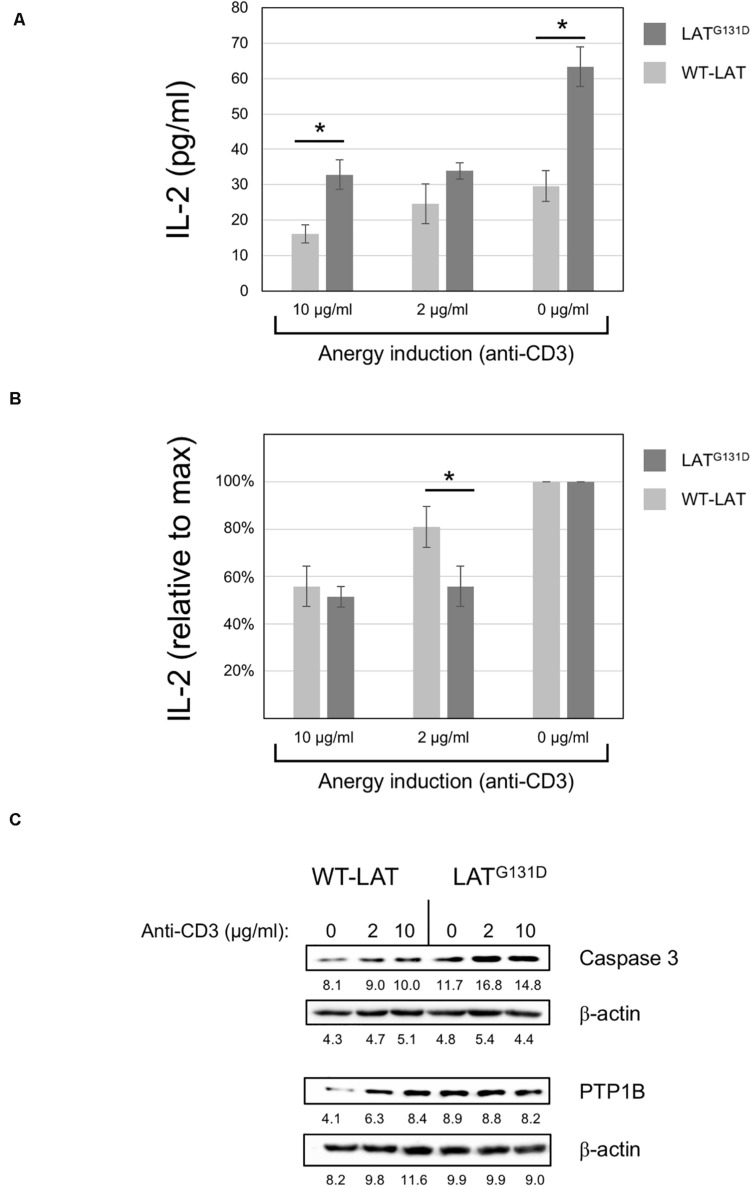
Anergy induction in J.CaM2 cells expressing the LAT^G131D^ mutant. **(A)** Anergy was induced in J.CaM2 cells expressing human WT-LAT or the LAT^G131D^ mutant by culturing cells overnight on plates with the indicated doses of the immobilized anti-CD3 antibody. Recovered cells were then stimulated with anti-CD3/CD28 microbeads for 48, and the amount of IL-2 in supernatants was measured by ELISA. The values presented are the mean values of four independent experiments. Bars represent the standard error. **(B)** The relative effect of anti-CD3 treatment on IL-2 production in cells expressing WT-LAT or the LAT^G131D^ mutant. Maximal IL-2 production in cells treated without anti-CD3 mAb (0 μg/ml) for each type of cell was considered 100%, and the relative production of IL-2 was calculated for each cell type and condition. **(C)** Caspase 3 and PTP1B levels in cells treated overnight with the indicated doses of immobilized anti-CD3 antibody were analyzed by Western blot (upper panels). Stripped membranes were blotted with anti-β-actin mAb to show equal protein expression (lower panels). The numbers below each panel represent the quantification of corresponding bands. Representative images from one of the three experiments performed with similar results.

It has been previously demonstrated that the integration of calciumsignals with activation of other signaling pathways results in fullactivation of T cells, while unopposed calcium signaling leads toanergy (Macian et al., 2002; Baine et al., 2009). Anergy is an essential mechanism of peripheral tolerance, established when the TCR is engaged in the absence of a CD28-mediated costimulatory signals. Evidence indicates that calcium signaling is responsible for the establishment of anergy in T cells. Given that J.CaM2 cells expressing the LAT^G131D^ mutant show increased PLC-γ activation and Ca^2+^ responses (Lo et al., 2019), it was of interest to study the relationship of this mutation with anergy. J.CaM2 or Jurkat cells are not the best models to analyze the anergy potential, because these cell lines proliferate continuously in a TCR independent way. However, we have shown that lentivirally transduced J.CaM2 cells can secrete IL-2 in response to CD3/CD28 stimulation, allowing us to study if anti-CD3 pretreatment has any effect on CD3/CD28-mediated IL-2 production. To do so, J.CaM2 cells lentivirally transduced to express WT-LAT or the LAT^G131D^ mutant were pretreated overnight with 2 or 10 μg/ml of immobilized anti-CD3 mAb, and then washed and stimulated with anti-CD3/CD28 microbeads for 48 h. IL-2 in the corresponding supernatants was then measured by ELISA. LAT^G131D^ expressing cells without anti-CD3 “anergizing” pretreatment (Figure 4A, 0 μg/ml of anti-CD3) produced 63 ± 6 pg/ml of IL-2, while WT-LAT expressing cells produced a statistically significant lower amount of IL-2 (30 ± 4 pg/ml). Overnight pretreatment of cells with 10 μg/ml of anti-CD3 reduced the amount of IL-2 produced by both types of cells by approximately half (16 ± 3 pg/ml for WT-LAT and 33 ± 4 pg/ml the LAT^G131D^, Figure 4A). Interestingly, pretreatment of WT-LAT expressing cells with a lower concentration of anti-CD3 (2 μg/ml) induced a modest decrease on IL-2 production by WT-LAT expressing cells (from 30 ± 4 pg/ml to 25 ± 6 pg/ml, Figure 4A), while the same treatment in LAT^G131D^ expressing cells still reduced by approximately half IL-2 production (from 63 ± 6 pg/ml to 34 ± 2 pg/ml, Figure 4A). Data in Figure 4A were recalculated to show the relative effect on the maximal IL-2 production of anti-CD3 pretreatment. Therefore, IL-2 secretion by both cell types at 0 μg/ml of anti-CD3 pretreatment were considered 100 percent of IL-2, and the amounts of IL-2 at 10 and 2 μg/ml of anti-CD3 were recalculated. As it can be seen in Figure 4B, pretreatment with 2 μg/ml of anti-CD3 reduced the IL-2 production by LAT^G131D^ expressing cells from 100% to 54% ± 4, while the same treatment had a reduced effect on WT-LAT expressing cells (from 100 to 84% ± 19). Therefore, these data demonstrate that pretreatment of LAT^G131D^ expressing cells with anti-CD3 antibodies produces a relative reduction in IL-2 secretion significantly greater than in cells expressing WT-LAT. The up-regulation of genes coding for caspase 3 and the phosphatase PTP1B has been previously correlated with anergy (Dominguez-Villar et al., 2007). To confirm the prediction that LAT^G131D^ mutant could have a pro-anergic effect, we analyzed caspase 3 and PTP1B expression by Western blot in cell lysates obtained after overnight treatment with 2 and 10 μg/ml of anti-CD3. As it can be seen in Figure 4C, J.CaM2 cells expressing the LAT^G131D^ mutant show increased basal levels of both caspase 3 and PTP1B with regard to WT-LAT expressing cells. Moreover, anti-CD3 stimulation also induced higher caspase 3 levels in LAT^G131D^ than in WT-LAT expressing cells, which endorses the pro-anergic effect of the Gly to Asp mutation of the amino acid preceding Tyr132 in LAT.

Overall, our data support previous results recently published by Arthur Weiss and co-workers (Lo et al., 2019). We have verified that cells expressing the LAT^G131D^ mutant show a significant and specific increase in LAT-Y132 phosphorylation. As a consequence, PLC-γ activation, Ca^2+^ influx generation, and Erk phosphorylation are augmented in LAT^G131D^ expressing cells after CD3-mediated stimulation. Interestingly, we have shown that this mutation, which prevents the binding to LAT of the specific mAb LAT-01, increases the stability of this protein. The increased LAT protein stability provided by the G131D mutation is in line with our previous published data (Arbulo-Echevarria et al., 2018). We have analyzed the role of a negatively charged segment of amino acids in LAT, which is very close to Gly 131. The substitution of this segment with a sequence of non-charged residues significantly decreased LAT stability. Now, we have demonstrated that introducing a negatively charged residue increases LAT stability. This increase in LAT stability could be related, at least in part, to the observed increase in activation events triggered after TCR engagement in LAT^G131D^ expressing cells. More experiments should clarify whether this is related to the introduction of a negative charge or to the removal of the glycine residue. Last, we have shown that substitution of glycine 131 by an aspartate residue enhances IL-2 production. Lo et al. have previously shown that this mutation lowers the reactivity threshold to allow for an increased percentage of cells producing IFN-γ, with notable differences when cells were stimulated with low-affinity peptides (Lo et al., 2019). Here we demonstrate that full activation of J.CaM2 cells expressing LAT^G131D^ mutant with anti-CD3/CD28 microbeads induces statistically significant greater amount of secreted IL-2. Moreover, we have shown that these cells show an enhanced predisposition to CD3-mediated inhibition of IL-2 production, which could be related to anergy in primary cells. This could be of immunological relevance, since to our knowledge, this is the earliest signaling event described to be related to anergy. Although Jurkat and J.CaM2 cell lines are not the best models for anergy studies, until *in vivo* analysis can be performed, our approach is a straightforward attempt to predict if anergy is affected by mutation of Gly 131. Other groups have analyzed different aspects of anergy using Jurkat cells (Tzachanis et al., 2001; Howe et al., 2003; Sundstrom et al., 2005; Dominguez-Villar et al., 2007; Li et al., 2010; Hsu et al., 2014; Liu et al., 2016; Sanchez-Del Cojo et al., 2017; Wang et al., 2019), which gives support to our experimental approach. The greater relative inhibition of IL-2 secretion shown by J.CaM2 cells expressing the LAT^G131D^ mutant supports this hypothesis, as it does the increased levels of caspase 3 and PTP1B. Future analysis of anergy induction in knockin mice expressing the same mutant LAT isoform would be useful to confirm these data, and this would constitute an invaluable model with which to analyze the role of anergy in the maintenance of tolerance and its implications for autoimmune disorders.

## Data Availability Statement

The original contributions presented in the study are included in the article/Supplementary Material, further inquiries can be directed to the corresponding author.

## Author Contributions

MA-E and EA designed the experiments. MA-E performed most of the experiments and interpreted results. IV-B and IN-S helped with cell culture, lentiviral transfections, and signaling experiments. MA-E, IV-B, FG-C, and AM provided input on study design and helped with manuscript writing. EA wrote the manuscript and directed the study. All authors contributed to the article and approved the submitted version.

## Conflict of Interest

The authors declare that the research was conducted in the absence of any commercial or financial relationships that could be construed as a potential conflict of interest.

## Supplementary Material

FIGURE S1 Expression of WT-LAT and LAT^G131D^ in J.CaM2 cells. **(A)** Schematic representation of the lentiviral vectors used to express WT-LAT and the LAT^G131D^ mutant in J.CaM2 cells. **(B)** Histograms of GFP and CD3 expression in lentivirally transduced J.CaM2 cells expressing WT-LAT (green line) or LAT^G131D^ mutant (black line). Numbers in the histograms represent the percentage of positive cells. **(C)** Non transduced J.CaM2 and J.CaM2 cells expressing WT-LAT or the LAT^G131D^ mutant were lysed and LAT expression was analyzed by Western blot with the anti-LAT mAb LAT-01 (left upper panel), anti-6His (middle upper panel), and the 11B.12 anti-LAT mAb (right upper panel). Membranes were stripped and blotted with anti-β-actin mAb to show equal protein load (lower panels).

FIGURE S2 Specific increase in TCR signaling in J.CaM2 cells expressing the LAT^G131D^ mutant. **(A)** Immunoblots analyzing phosphorylation of LAT at tyrosine residue 171 in cells stimulated with soluble anti-CD3 were done with phospho-specific antibody. Equal amounts of the same samples were run in parallel and analyzed for total LAT expression by Western blot (lower panel). Numbers below each panel represent quantification of corresponding bands. Representative images from one of the three experiments performed with similar results. **(B)** Western blot analysis of PLC-γ activation (upper panel). Membranes were stripped and blotted with anti-β-actin mAb to show equal protein load (lower panel). Numbers below each panel represent quantification of corresponding bands. Representative images from one of the three experiments performed with similar results. **(C)** J.CaM2 cells expressing WT-LAT or the LAT^G131D^ mutant were loaded with Indo-1AM and stimulated with the indicated concentrations of anti-CD3 mAb at the indicated time (black arrows). The intracellular Ca^2+^ concentration was determined at 37°C through the change in Indo-1AM fluorescence. Graphs represent the average of 3 and 5 experiments, for OKT3 concentrations of 0.5 and 0.125 μg, respectively.

FIGURE S3 Stable expression of LAT after long-term CD3-stimulation. Immunoblots analyzing expression of LAT (upper panel) and β-actin (lower panel) in cells treated overnight with the indicated doses of immobilized anti-CD3 antibody. Molecular weights in kDa are indicated on the side of the upper panel.
