# Supplementary material for: Increased Protein Stability and Interleukin-2 Production of a LATG131D Variant With Possible Implications for T Cell Anergy
Source: Front Cell Dev Biol. 2020 Sep 11;8:561503. doi: 10.3389/fcell.2020.561503 (PMC7517355; doi:10.3389/fcell.2020.561503)
Supplement: FIGURE S1 — Expression of WT-LAT and LATG131D in J.CaM2 cells. (A) Schematic representation of the lentiviral vectors used to express WT-LAT and the LATG131D mutant in J.CaM2 cells. (B) Histograms of GFP and CD3 expression in lentivirally transduced J.CaM2 cells expressing WT-LAT (green line) or LATG131D mutant (black line). Numbers in the histograms represent the percentage of positive cells. (C) Non transduced J.CaM2 and J.CaM2 cells expressing WT-LAT or the LATG131D mutant were lysed and LAT expression was analyzed by Western blot with the anti-LAT mAb LAT-01 (left upper panel), anti-6His (middle upper panel), and the 11B.12 anti-LAT mAb (right upper panel). Membranes were stripped and blotted with anti-β-actin mAb to show equal protein load (lower panels). [file Data_Sheet_1.pdf]

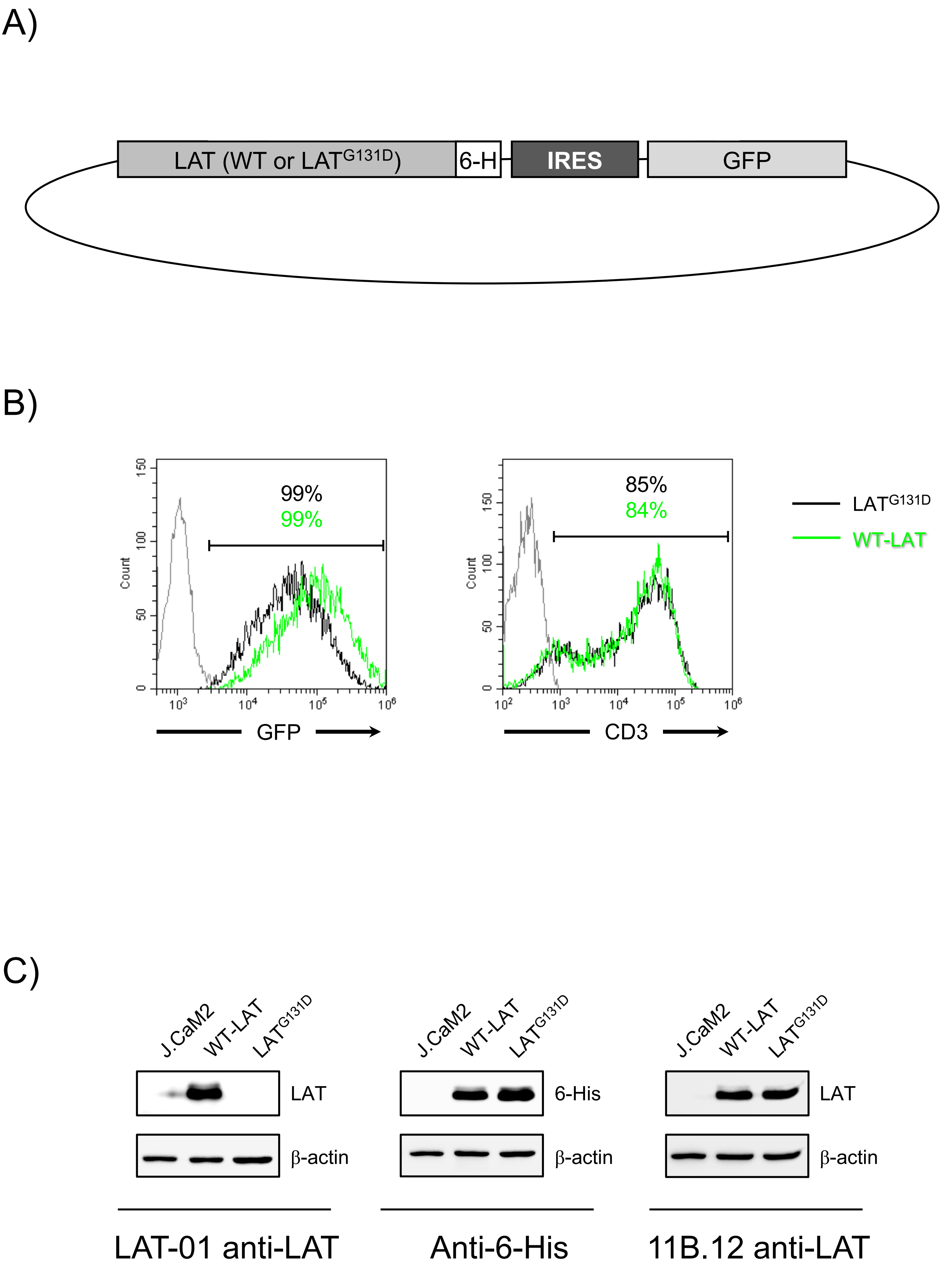

Supplementary Figure 1

Supplementary Figure 1. Expression of WT-LAT and LAT^{G131D} in J.CaM2 cells. A) Schematic representation of the lentiviral vectors used to express WT-LAT and the LAT^{G131D} mutant in J.CaM2 cells. B) Histograms of GFP and CD3 expression in lentivirally transduced J.CaM2 cells expressing WT-LAT (green line) or LAT^{G131D} mutant (black line). Numbers in the histograms represent the percentage of positive cells. C) Non transduced J.CaM2 and J.CaM2 cells expressing WT-LAT or the LAT^{G131D} mutant were lysed and LAT expression was analyzed by Western blot with the anti-LAT mAb LAT-01 (left upper panel), anti-6His (middle upper panel), and the 11B.12 anti-LAT mAb (right upper panel). Membranes were stripped and blotted with anti-β-actin mAb to show equal protein load (lower panels).

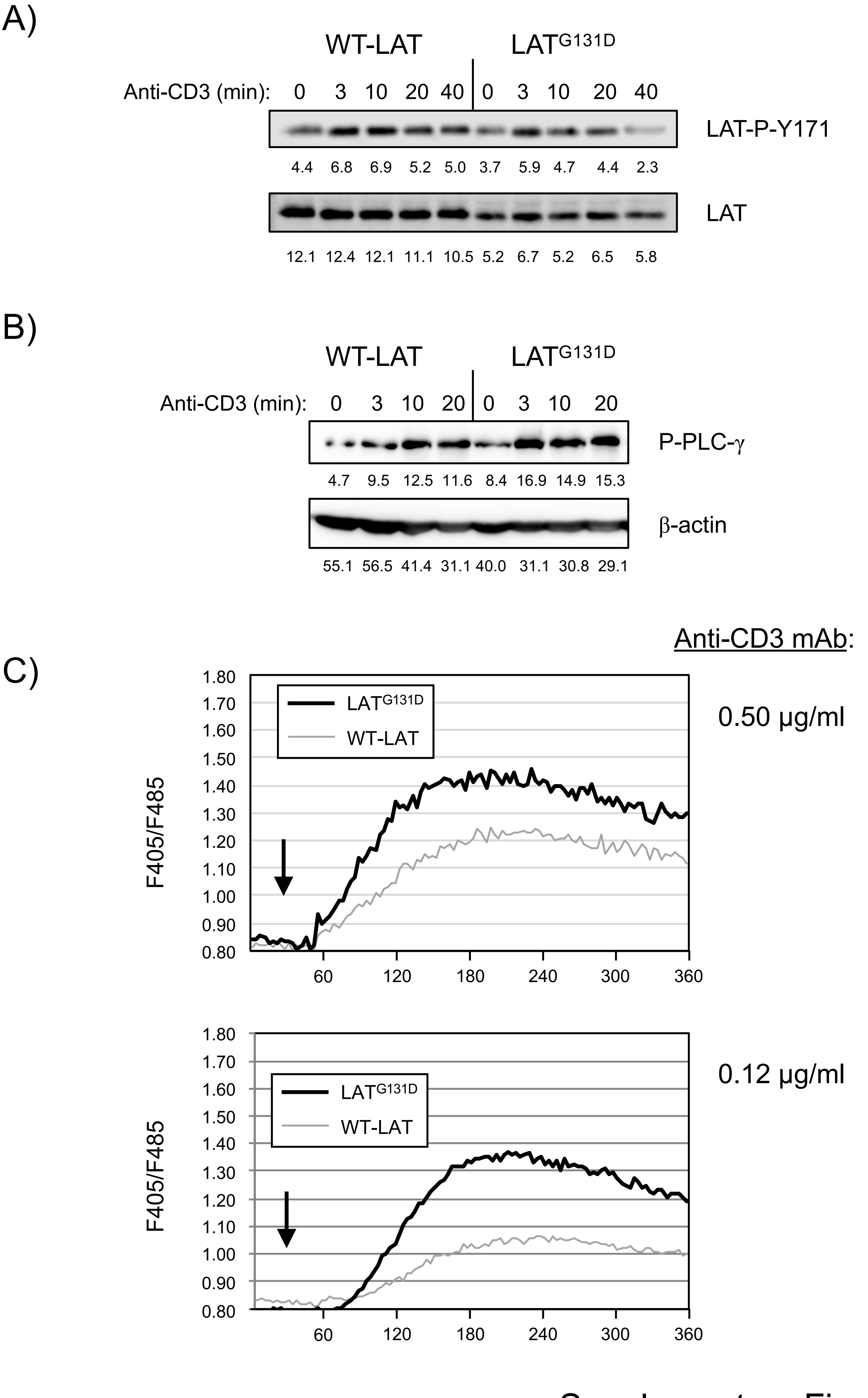

Supplementary Figure 2

Supplementary Figure 2. Specific increase in TCR signaling in J.CaM2 cells expressing the LAT^{G131D} mutant. A) Immunoblots analyzing phosphorylation of LAT at tyrosine residue 171 in cells stimulated with soluble anti-CD3 were done with phospho-specific antibody. Equal amounts of the same samples were run in parallel and analyzed for total LAT expression by Western blot (lower panel). Numbers below each panel represent quantification of corresponding bands. Representative images from one of the three experiments performed with similar results. B) Western blot analysis of PLC-γ activation (upper panel). Membranes were stripped and blotted with anti-β-actin mAb to show equal protein load (lower panel). Numbers below each panel represent quantification of corresponding bands. Representative images from one of the three experiments performed with similar results. C) J.CaM2 cells expressing WT-LAT or the LAT^{G131D} mutant were loaded with Indo-1AM and stimulated with the indicated concentrations of anti-CD3 mAb at the indicated time (black arrows). The intracellular Ca^{2+} concentration was determined at 37°C through the change in Indo-1AM fluorescence. Graphs represent the average of 3 and 5 experiments, for OKT3 concentrations of 0.5 μg and 0.125 μg, respectively.

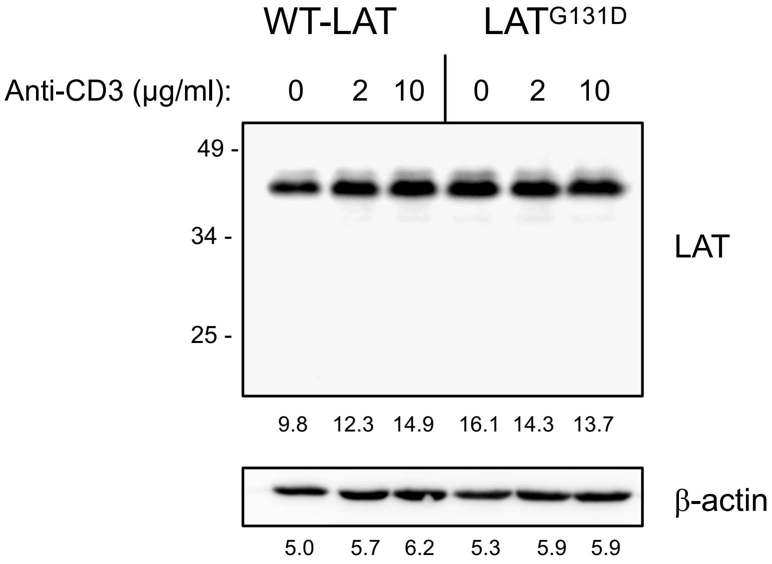

WT-LAT
LAT G131D
Anti-CD3 (µg/ml): 0 2 10 0 2 10
49 -
34 -
25 -
LAT
9.8 12.3 14.9 16.1 14.3 13.7
β-actin
5.0 5.7 6.2 5.3 5.9 5.9

Supplementary Figure 3. Stable expression of LAT after long-term CD3-stimulation. Immunoblots analyzing expression of LAT (upper panel) and β-actin (lower panel) in cells treated overnight with the indicated doses of immobilized anti-CD3 antibody. Molecular weights in kDa are indicated on the side of the upper panel.
